# Melatonin treatment has consistent but transient beneficial effects on sleep measures and pain in patients with severe chronic pain: the DREAM–CP randomised controlled trial

**DOI:** 10.1016/j.bja.2024.01.012

**Published:** 2024-02-14

**Authors:** Uzunma M. Onyeakazi, Malachy O. Columb, Adam Rosalind, Saravanakumar Kanakarajan, Helen F. Galley

**Affiliations:** 1 School of Medicine, Medical Sciences and Nutrition, University of Aberdeen, Aberdeen, UK; 2 Manchester University Hospitals NHS Foundation Trust, Wythenshawe, UK

**Keywords:** chronic pain, clinical trial, crossover, melatonin, sleep

## Abstract

**Background:**

Sleep disturbance is a major issue for patients with chronic pain. Melatonin has been shown to improve symptoms of fibromyalgia, but its efficacy in other chronic non-malignant pain conditions is not fully known. Hence, we determined the effect of melatonin in patients with severe noncancer chronic pain.

**Methods:**

This was a randomised double-blinded crossover trial of modified-release melatonin as Circadin™ compared with placebo. Sixty male and female subjects with chronic severe pain were randomised to receive either 2 mg of Circadin™ or placebo before sleep for 6 weeks, followed by a >4 week washout, then crossing over to the other treatment. Sleep disturbance, quality, and latency were measured using three different validated sleep assessment tools. The primary outcome measure was self-reported sleep disturbance after 6 weeks of treatment. Adverse events were also recorded.

**Results:**

Sleep disturbance after 6 weeks was not significantly altered by melatonin treatment, but differences between melatonin and placebo treatment periods after 3 weeks were seen: sleep disturbance (*P*=0.014), latency (*P*=0.04), overall sleep quality (*P*=0.004), and effect of pain on sleep (*P*=0.032). Pain intensity scores improved during both treatment periods (both *P*<0.001). There were no differences in adverse events between treatment periods.

**Conclusions:**

Circadin™ treatment did not improve sleep disturbance in patients with severe chronic pain compared with placebo at 6 weeks, but there were consistent improvements in aspects of sleep in the shorter term. Given its favourable safety profile, it could be beneficial for some patients with chronic pain.

**Clinical trial registration:**

ISRCTN12861060.

**Editor's key points**

• Sleep disorders are associated with chronic pain in up to two-thirds of cases.
• Melatonin, an endogenous hormone, has a regulatory role in the sleep–wake cycle, but its beneficial effects on sleep in patients with chronic pain are not fully clarified.
• In this randomised, double-blinded, placebo-controlled crossover trial investigating for the first time a licenced formulation of melatonin (Circadin™) in patients with severe chronic non-malignant pain, melatonin did not improve sleep disturbance at 6 weeks but improved sleep parameters at 3 weeks.
• Considering its excellent safety profile, melatonin holds promise for individual chronic pain patients with flare-up episodes or in need of respite.

Sleep is one of the important domains in managing chronic pain. Sleep disturbance increases the suffering, disability, and perception of pain.^1^ A recent meta-analysis found pooled prevalence of sleep disturbances in chronic non-cancer pain as high as 75.9%.^2^ In addition to the neuronal mechanisms, the neurobiology of pain mechanisms implicates the involvement of non-neuronal components such as opioids, monoaminergic, endocannabinoids, immune system, and melatonin.^3^ These non-neuronal components are involved in regulation of multiple other systems including the sleep–wake cycle. Lack of sleep and poor sleep quality have been found to be a risk factor for the development of chronic pain by epidemiological studies, and experimental studies have found that sleep disturbance not only increases sensitivity to pain but also exaggerates pain symptoms.^4^ Thus, there is a bidirectional association between sleep and pain.^5^

Melatonin, an endogenous hormone secreted mainly by the pineal gland in response to low light levels, has a regulatory role in the sleep–wake cycle. In addition, it has other properties including anti-inflammatory effects.^6^^,^^7^ Melatonin can be easily synthesised chemically and is available for exogenous administration in several formulations. However, the only licensed melatonin product in the UK is a modified-release form of melatonin called Circadin™ which is licensed at a dose of 2 mg for the treatment of primary insomnia in people aged over 55 yr.^8^^,^^9^

In animal models, exogenous melatonin has been found to have beneficial effects on nociception, hyperalgesia, allodynia, and pain behaviour.^10^^,^^11^ In people with fibromyalgia, melatonin treatment was reported to reduce scores in the fibromyalgia impact questionnaire.^12^ Several studies have also evaluated the effect of melatonin on pain and anxiety in the perioperative period, but a systematic review and meta-analysis found that overall data were unreliable because of the marked heterogeneity.^13^

In our earlier study of sleep assessment tools, we found that sleep disturbance and poor sleep quality correlated with pain intensity in patients with chronic non-cancer pain.^14^ We hypothesised that administration of exogenous melatonin would reduce sleep disturbance in patients with severe chronic pain and improve pain. This paper presents the results of a double-blinded, placebo-controlled, randomised, crossover trial evaluating the efficacy of oral melatonin as Circadin™ in patients with severe chronic non-malignant pain.

## Methods

### Trial design

The study was classed as a Clinical Trial of an Investigational Medicinal Product (CTIMP), and a clinical trial authorisation was obtained from the Medicines and Healthcare products Regulatory Authority (MHRA) in the UK, in addition to a favourable ethical opinion from the Office for Research Ethics Committees Northern Ireland (reference 19/NI/0007). Participants were recruited between 2019 and 2022. The protocol for this study has been published,^15^ although some minor modifications to trial processes were required during the COVID-19 pandemic. The trial was a single-centre, double-blinded, randomised, placebo-controlled crossover design and was prospectively registered at https://www.isrctn.com/ISRCTN12861060.

Trial Steering and Data and Safety Monitoring Committees with external chairs were established. The study was sponsored jointly by the University of Aberdeen and NHS Grampian and was monitored by NHS Grampian. The work was funded by the *British Journal of Anaesthesia*/Royal College of Anaesthetists via the National Institute of Academic Anaesthesia (reference number WKRO-2017-0043), and Circadin™ and placebo were provided by Flynn Pharma Ltd (Stevenage, UK).

### Recruitment

After written informed consent, patients attending a pain management clinic at a tertiary referral teaching hospital in Northeast Scotland were recruited. Participants had severe chronic pain for at least 3 months, defined as an average pain intensity score of at least 7 (on an 11-point numerical rating scale) using the Brief Pain Inventory (BPI). Patients were excluded if they had malignant pain; were under 18 yr of age; had a history of liver dysfunction or alcohol or drug abuse; or were pregnant, breastfeeding, or planning to get pregnant. Patients taking nifedipine or fluvoxamine, benzodiazepines, or non-benzodiazepine hypnotics (zaleplon, zolpidem, and zopiclone) were also excluded owing to their interaction with melatonin metabolism or impact on sleep. After initial screening by the clinical care team, eligible potential participants were sent letters of invitation and a participant information sheet ahead of a scheduled clinic visit. The researcher met with potential participants (either in person or by secure video call during the pandemic) who had expressed interest in the trial, explained the study, and obtained written consent (witnessed by secure video call during the pandemic).

### Trial medication and randomisation

Circadin™ is a modified-release formulation of melatonin with a blood concentration profile resembling that of endogenous melatonin. Both Circadin™ and an identical placebo were provided free of charge by Flynn Pharma and were repackaged, labelled, and dispensed by the Clinical Trials Pharmacy at Aberdeen Royal Infirmary, who were also responsible for drug accountability. Flynn Pharma did not have any input to trial conduct. Participants were randomised in blocks of 10 to receive either 2 mg of Circadin™ or a placebo of identical appearance for 6 weeks, then the opposite for another 6 weeks, with a washout period in-between of at least 4 weeks. Participants were instructed to take medication at around 20:00 daily and to refrain from consuming alcohol throughout the trial. The randomisation schedule was provided to the Clinical Trials Pharmacy in advance by an independent external statistician not involved in the trial. All participants, research staff, and the statistician were blinded to allocation until after data analysis.

### Participant visits

**Fig 1:**
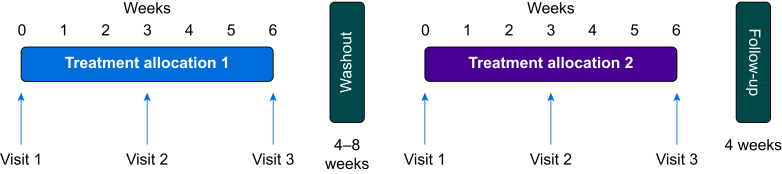
Schematic view of the trial design showing the ‘journey’ that each participant followed. For Group A, treatment allocation 1 was melatonin and treatment allocation 2 was placebo. For Group B, treatment allocation 1 was placebo and treatment allocation 2 was melatonin.

Participants attended a baseline visit, then two visits 3 weeks apart during each treatment period, and a follow-up visit 4 weeks after the last treatment period ended (Fig. 1). During the COVID-19 pandemic, these visits were undertaken by secure video call using ‘NHS NearMe’, with drugs and paperwork delivered to participants' homes by researchers following a strict protocol to ensure the safety of both participant and researcher. Medication containers were collected, and tablet counts were used to assess compliance. At each visit, the participants completed three sleep scales: the Verran and Snyder–Halpern (VSH) scale,^14^^,^^16^^,^^17^ which assesses sleep over the previous 24 h; the Pittsburgh Sleep Quality Index (PSQI),^14^^,^^18^ which records sleep quality over the last month; and the Pain and Sleep Questionnaire three-item index (PSQ-3),^14^^,^^19^ which evaluates the impact of pain on sleep over the previous week. In addition, the BPI was completed at each visit. Participants wore a Philips Actiwatch Spectrum Pro (Linton Instrumentation, Norfolk, UK) constantly throughout the treatment periods and inputted pain and fatigue scores at 08:00 and 20:00 daily in response to an automated reminder alarm. At the end of participation, participants were given a post-trial questionnaire to fill out anonymously to evaluate the trial from their perspective. Participants were questioned weekly regarding adverse events (AEs).

### Trial outcome measures

The primary outcome measure was VSH sleep disturbance after 6 weeks of treatment. Secondary outcomes were sleep disturbance at 3 weeks, plus sleep quality global score using PSQI, impact of pain on sleep using PSQ-3, and pain intensity and sleep interference scores using the BPI, at 3 and 6 weeks, along with twice-daily pain and fatigue scores inputted to an Actiwatch.

### Melatonin measurement and reaction time testing

Serum melatonin measurement and psychomotor vigilance testing^17^^,^^20^ were planned to monitor melatonin ‘hangover’ and any daytime drowsiness. However, COVID-19 pandemic restrictions meant that data were only available from the first 18 participants. Venous blood was collected in an 8.5 ml BD Vacutainer SST Advance Tube (Fisher Scientific UK Ltd., Loughborough, UK.) using a closed Vacutainer system. The blood was then centrifuged, and the serum stored at –80°C until assay. Serum melatonin was measured in-house using a commercially available melatonin competitive binding enzyme-linked immunosorbent assay kit (Abbexa Ltd., Cambridge, UK).

### Sample size and statistical analysis

The primary outcome measure was VSH sleep disturbance. Our previous data showed that patients with mild/severe pain had median VSH sleep disturbance scores of 147 and 490, respectively (maximum possible score: 700).^14^ Assuming a conservative treatment effect difference of 120 (effect size: 0.60), we calculated that a minimum of 46 patients would be needed to achieve 80% power using a crossover design. It was therefore aimed to recruit 60 participants to allow for withdrawals, to achieve at least 46 patients completing both treatment periods. Data were analysed as intention-to-treat (ITT), per protocol, and treatment-received, using linear mixed models. Crossover analyses included tests for the effects of treatment, period, treatment–period interaction (carryover effect), and sequence. Baseline measures were entered as covariates. Bonferroni corrections were applied for multiple comparisons as appropriate. Data were analysed using Number Cruncher Statistical Systems (NCSS) version 2020 (NCSS Inc., Kaysville, UT, USA) and Stata 17.0 (Stata Inc., College Station, TX, USA). Significance was defined at *P*<0.05 (two-sided). Data are presented as median, inter-quartile, and full range.

## Results

**Fig 2:**
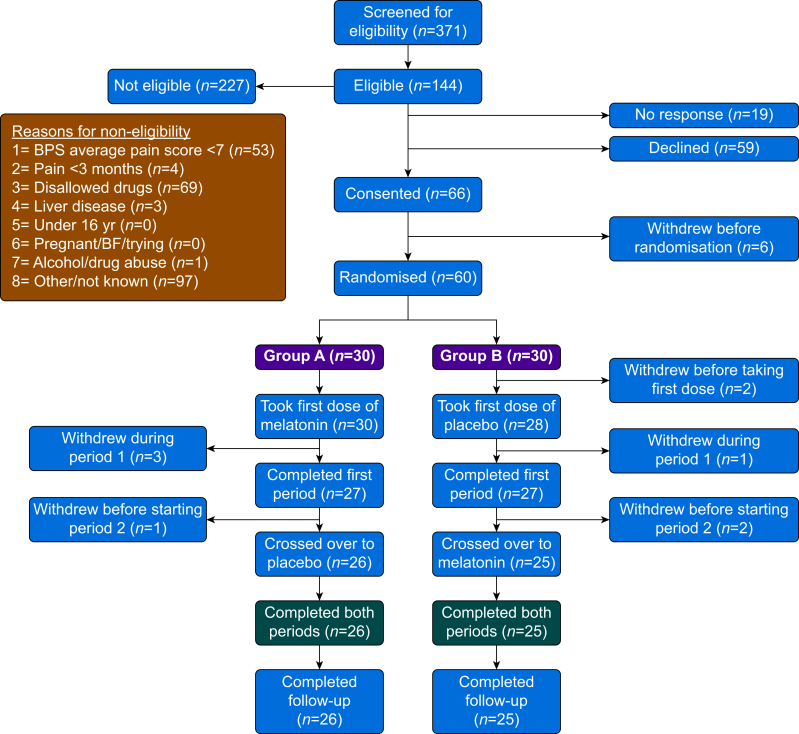
CONSORT diagram for randomised crossover trials.

Recruitment is summarised in the CONSORT diagram in Figure 2. Between June 2019 and October 2021, 371 patients were screened for eligibility and 66 eligible participants consented to take part; six of these withdrew before randomisation and were not included in the ITT analysis. Of the 58 participants who received at least one dose of trial medication and were included in the ITT analysis, 30 were allocated to receive melatonin first (Group A) and 28 to receive placebo first (Group B). A total of 51 participants completed as per protocol, with 26 of these randomised to receive melatonin first and 25 to receive placebo first. All participants received their allocated treatment as randomised, such that the per protocol and treatment-received analyses are identical. Median compliance based on returned tablet count was 96 (40–100)%. We present the ITT analysis.

### Baseline characteristics

Table 1 shows the baseline characteristics of participants by ITT and per protocol analysis. Participant groups were similar with respect to age, sex, pain duration, and BMI. The majority of participants had a BMI within the obese range (BMI: ≥30). There were no significant differences in sampling probabilities of the outcome measures at baseline between groups (Table 2). Most participants were taking several different medications and these are summarised in Supplementary Table S1.

**Table 1:** Characteristics of participants at baseline. Data are shown as number or median (range) as appropriate, with % in parentheses.

	Group A	Group B
	Melatonin first (*n*=30)	Placebo first (*n*=28)
**Age (yr), %**	62 (24–79)	55 (28–79)
**Sex, *n* (%)**		
Male	10 (33)	12 (43)
Female	20 (67)	16 (58)
**Pain duration (months)**	96 (12–336)	96 (24–480)
**Pain location, *n* (%)**		
Neck	3 (10)	1 (4)
Thorax/upper back/abdomen	2 (7)	3 (11)
Upper or lower limbs	7 (23)	10 (36)
Lower back	7 (23)	9 (32)
Widespread	11 (37)	5 (418)
**Pain type, *n* (%)**		
Nociceptive/mainly nociceptive	15 (50)	1 (39)
Neuropathic/mainly neuropathic	7 (23)	11 (39)
Mixed	8 (27)	6 (21)
**Ethnicity, *n* (%)**		
White	28 (93)	27 (96)
Other White	1 (3)	0
Asian	1 (3)	0
Black	0	1 (4)
**Smoking status, *n* (%)**		
Never smoked	10 (33)	15 (55)
Ex-smoker	13 (43)	9 (32)
Smoker	7 (23)	4 (14)
**BMI (kg m^−2^)**	29.8 (22.7–51.3)	30.3 (20.4–48.0)
**BMI categories, *n* (%)**		
Normal (18.5–24.9)	3 (10)	4 (14)
Overweight (25.0–29.9)	12 (40)	9 (32)
Obese (≥30)	14 (47)	15 (54)
Missing value	1 (3)	0

**Table 2:** Baseline outcome measure data (intention-to-treat). Data shown as median (range). ∗Range 0–700. ^†^Range 0–100. ^‡^Inclusion criteria dictate score of ≥7 at baseline. ^¶^Range 0–10. ^§^Range 0–30. ^||^Range 0–21. ^#^Range 0–300.

	Group A	Group B	Sampling probability (*P*-value)
	Melatonin first	Placebo first	
**Verran and Snyder–Halpern sleep scale**			
Sleep disturbance∗	481 (67–632)	409 (122–673)	0.06
Sleep latency^†^	89 (4–100)	50 (0–100)	0.18
Wake after sleep onset^†^	54 (3–100)	43 (0–90)	0.24
**Brief Pain Inventory**			
Pain intensity score^‡,¶^	7 (7–10)	7 (7–10)	0.38
Sleep interference score^¶^	8 (1–10)	8 (5–10)	0.86
Psychological interference score^§^	17 (0–30)	17 (3–28)	0.75
**Pittsburgh Sleep Quality Index**			
Global score^||^	12.5 (4.0–17.0)	12.0 (5.0–16.0)	0.31
Sleep duration (h)	5.0 (2.0–7.5)	2.0 (2.0–8.0)	0.56
**Pain and Sleep Quality three-item score**^#^	248 (57–300)	214 (83–299)	0.08

#### Verran and Snyder–Halpern sleep scale

**Fig 3:**
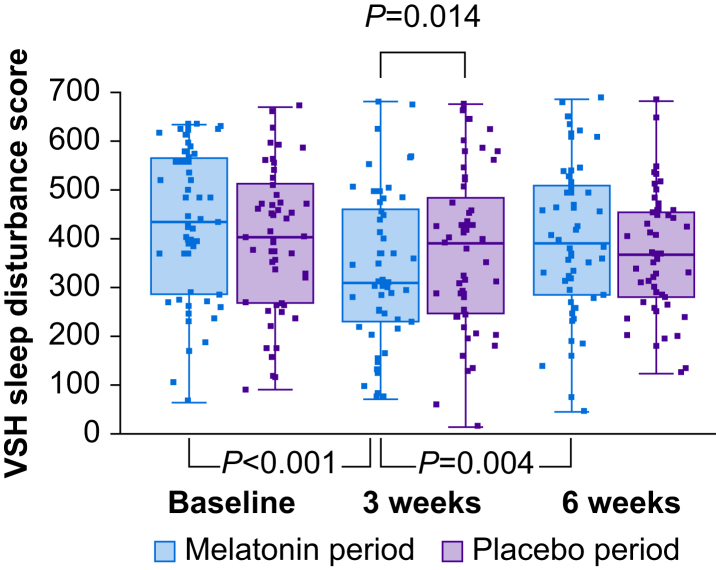
Verran and Snyder–Halpern (VSH) sleep disturbance scores (intention-to-treat analysis). There was a significant difference between melatonin and placebo treatment periods at Visit 2 (3 weeks, *P*=0.014) but not at Visit 3 (6 weeks). There was also a significant difference between Visit 1 (baseline) and Visit 2 (3 weeks, *P*<0.001) and between Visit 2 and Visit 3 (*P*=0.004) during melatonin but not placebo treatment periods. Box and whisker plots show median, inter-quartile, and full range with individual data points overlaid. Linear mixed effects models for treatment (*P*=0.13), sequence (*P*=0.99), and period (*P*=0.66) were not significant. *P*-values shown are Bonferroni corrected as appropriate.

Baseline sleep disturbance scores were high in both groups with median (range) baseline scores of 481 (67–632) in Group A and 409 (122–673) in Group B (Table 2). The primary outcome measure of sleep disturbance as described by the VSH sleep scale at 6 weeks was not significantly different between the melatonin and placebo treatment periods (Fig. 3). However, significant decreases in VSH sleep disturbance scores were seen after 3 weeks of melatonin treatment (*P*<0.001) which were not seen during placebo treatment, such that there was a significant difference at 3 weeks between the two treatment periods (*P*=0.014, Fig. 3). Likewise, other sleep measures captured by the VSH scale also showed decreases after 3 weeks of melatonin treatment, including sleep latency score (the time taken to fall asleep, *P*<0.001) and wake after sleep onset (WASO, *P*=0.024), compared with baseline. There was also a between-treatment difference for sleep latency at 3 weeks (*P*=0.004, Supplementary Fig. S1).

#### Pain and Sleep Questionnaire three-item index

**Fig 4:**
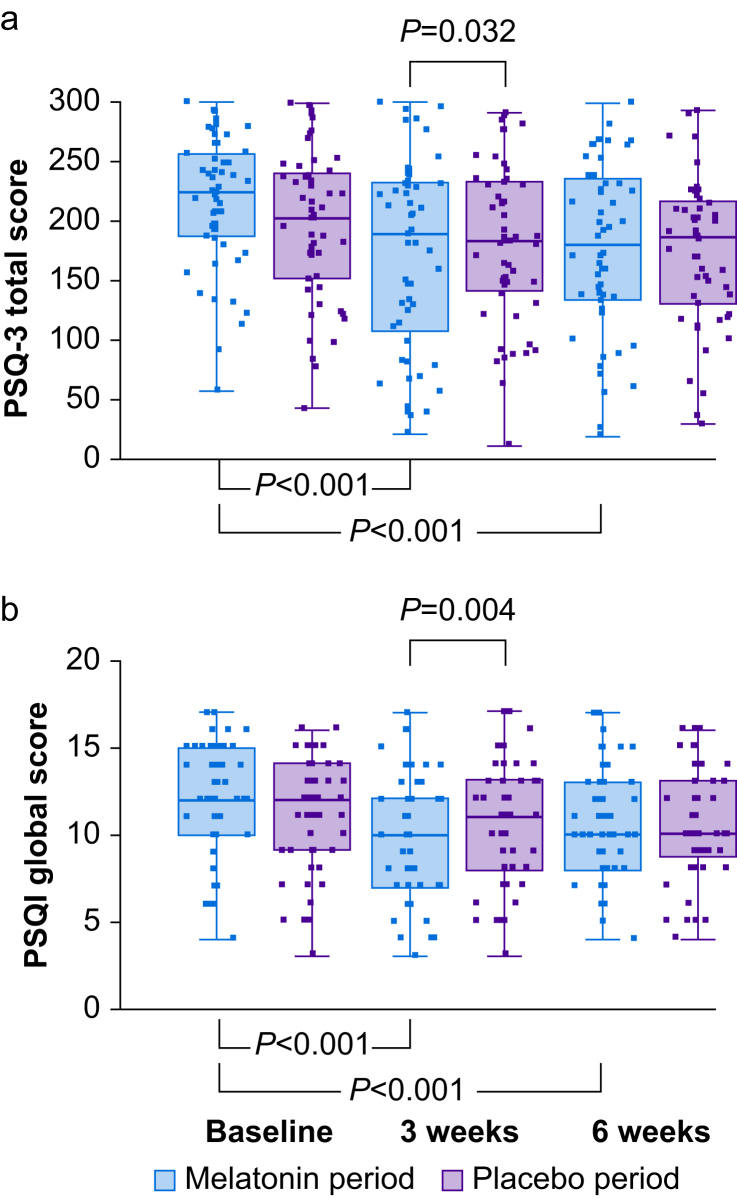
(a) Pain and Sleep Questionnaire three-item index (PSQ-3) (intention-to-treat analysis). There was a significant difference in PSQ-3 between melatonin and placebo treatment periods at Visit 2 (3 weeks, *P*=0.032) but not at Visit 3 (6 weeks). There was also a significant difference in PSQ-3 between Visit 1 (baseline) and Visit 2 (*P*<0.001) and between Visit 2 and Visit 3 (*P*<0.001) during melatonin but not placebo treatment periods. (b) Pittsburgh Sleep Quality Index (PSQI) global sleep quality score (intention-to-treat analysis). There was a significant difference in PSQI between melatonin and placebo treatment periods at Visit 2 (3 weeks, *P*=0.004) but not at Visit 3 (6 weeks). There also was a significant difference in PSQI between Visit 1 (baseline) and Visit 2 (*P*<0.001) and between Visit 2 and Visit 3 (*P*<0.001) during melatonin but not placebo treatment periods. Box and whisker plots show median, inter-quartile, and full range with individual data points overlaid. Linear mixed effects models on PSQ-3 were significant for treatment (15.3; 95% confidence interval [CI] 1.1–29.5; *P*=0.034), but not for sequence (*P*=0.51) and period (*P*=0.38). Linear mixed effects models on PSQI were significant for treatment (0.74; 95% CI 0.16–1.32; *P*=0.012), but not for sequence (*P*=0.35) and period (*P*=0.53). *P*-values shown are Bonferroni corrected as appropriate.

The median (range) baseline PSQ-3 score was 248 (57–300) in Group A and 214 (83–299) in Group B (Table 2), indicating pain-related sleep issues. Compared with baseline, there were significant decreases in PSQ-3 score after 3 and 6 weeks of melatonin treatment (both *P*<0.001). A significant difference was noted between PSQ-3 scores after 3 weeks' melatonin compared with placebo treatment (*P*=0.032, Fig. 4a).

#### Pittsburgh sleep quality index

The median (range) PSQI global score at baseline was 12 (4–17) in Group A and 12 (5–16) in Group B (Table 2). A global quality score of >5 indicates poor sleep quality; only one participant had a score below 5. Global sleep quality scores increased significantly after 3 and 6 weeks of melatonin treatment (*P*<0.001) with a significant difference between melatonin and placebo treatment periods at 3 weeks (*P*=0.004, Fig. 4b). Self-reported sleep duration captured by the PSQI was not different between treatment periods at 6 weeks (5.5 [0–8] h and 5.5 [1–8] h after melatonin or placebo, respectively) and no change within groups was seen during either treatment.

#### Brief Pain Inventory

Average pain intensity captured by the BPI decreased significantly during both melatonin and placebo treatment periods (both *P*<0.001, Supplementary Fig. S2). Sleep interference scores were similar at baseline in both groups (Table 2) and decreased during both melatonin and placebo treatment (both *P*<0.001). Interference scores of psychological parameters (mood, relations with other people, and enjoyment of life) did not change.

#### Actiwatch data

Twice-daily ‘actual pain now’ scores inputted to the Actiwatch showed a very small but significant overall reduction during melatonin treatment compared with placebo treatment (0.24; 95% confidence interval [CI] 0.08–0.39; *P*=0.003; Supplementary Table S2). Likewise, fatigue scores, also entered twice daily into the Actiwatch, also showed a small but significant reduction with melatonin treatment (0.20; 95% CI 0.03–0.38; *P*=0.025; Supplementary Fig. S3). Sleep duration captured by the Actiwatch was around 7.5 h, ranging from 2.2 h to 21.6 h, but some of the data values seem implausible (Supplementary Table S2). No statistically significant differences were found, and the data did not reflect the findings from other sleep assessment tools.

### Melatonin analysis

The original intention was to measure circulating melatonin concentration in all participants at every visit, 12 h after the last dose of trial drug was taken, to ensure there was no hangover effect likely to cause daytime drowsiness, as we have previously reported with 6 mg doses of Circadin™.^17^ However, COVID-19 restrictions meant that blood samples were only able to be obtained from the first 18 participants. There was no difference in circulating melatonin concentration during the melatonin or placebo treatment and no change during treatments (Supplementary Fig. S4) in this subset of participants.

### Per protocol analysis

The per protocol and ITT results were similar; there was no difference between melatonin and placebo treatment periods in VSH disturbance (Supplementary Fig. S5) or other sleep parameters at 6 weeks (Supplementary Table S4), but all scores were significantly different at 3 weeks as for the ITT analysis. Analysis by treatment received was not performed as there were no treatment crossovers and all participants received the treatment they had been randomised to receive.

### Adverse events

Two serious adverse events (SAEs) occurred in two participants allocated to Group B during the washout period (i.e. after placebo treatment). One SAE was acute appendicitis and the other was exacerbation of existing asthma, both resulting in hospital admission and hence their classification as SAEs. Both were considered to be unrelated to trial drug treatment.

There were a total of 116 AEs in 46 participants recorded during the trial. The total was not significantly different during melatonin and placebo treatment periods (49 and 47, respectively). Of these, 19 events occurred during the washout period and one during follow-up (Supplementary Table S4). Of the 19 events, 12 (10.3%) were considered to be possibly related to melatonin treatment at the time of reporting the event. Expected AEs during melatonin administration are drowsiness, headache, and nausea. Headaches were more commonly reported during the placebo period and there were three separate reports of drowsiness: one in Group A during melatonin treatment and one in the follow-up period after melatonin treatment, and one in Group B during placebo treatment (Supplementary Table S4). There were four reports of nightmares in three different participants. Three of these were during the melatonin treatment period and one during placebo treatment. One of the participants unilaterally decided to discontinue treatment after a second nightmare event which occurred during the melatonin treatment period. AEs classified as gut-related were reported a similar number of times during melatonin treatment (*n*=10) as during placebo (*n*=9) by different participants. Skin issues were reported by six participants; four of these were caused by rashes under the rubber Actiwatch strap, linked to strap tightness and sweating, which was treated with topical hydrocortisone and extension of the strap where necessary. There was no trend to the AEs reported during follow-up after trial drug treatment had stopped. All of the AEs were mild and transient and gave no cause for concern.

### Post-trial questionnaire

The response rate to the anonymous post-trial questionnaire was 86%. All responders strongly agreed/agreed that the trial was well organised, and that communication was good. Only 7% strongly agreed/agreed that they had side-effects from the treatment and 90.6% strongly disagreed/disagreed. Twenty-eight (62.2%) of the participants who responded agreed/strongly agreed they would take melatonin if it were available, 11 (24.4%) were unsure whether they would or not, and six (13.3%) indicated that they would not take it.

## Discussion

We report here a single-centre, randomised, double-blinded, placebo-controlled, crossover trial of melatonin as Circadin™ in patients with severe chronic non-malignant pain. We found that melatonin treatment did not improve sleep disturbance at 6 weeks but was associated with improved sleep parameters captured with all three sleep assessment tools at 3 weeks. Adverse events were similar during both melatonin and placebo treatment periods.

Administration of melatonin reduces sleep latency and other sleep measures in older adults with primary insomnia, but not everyone responds to melatonin, with absolute responder rates of between 27% and 42%.^8^ Circadin™ is given for up to 12 months to treat primary insomnia, with no rebound or withdrawal effects and no apparent loss of efficacy over time.^8^ Melatonin has been proposed to not only help sleep in patients with chronic pain but also improve pain.^3^^,^^21^

To the best of our knowledge, this is the first study of a licensed formulation of melatonin (Circadin™) in patients with severe chronic non-malignant pain of varied aetiology. The analgesic effect of melatonin has been evaluated in patients with specific pain-related conditions (fibromyalgia, endometriosis, and irritable bowel syndrome), often in combination with other therapies, with mixed results. In endometriosis-associated pelvic pain, a group in Brazil reported improvements in pain after 8 weeks of treatment with 10 mg of melatonin.^22^ However, a very recently published similar study in patients with endometriosis pain failed to show any effect of 20 mg melatonin after 8 weeks of treatment.^23^ The Brazilian group have also reported beneficial effects of different doses of melatonin on pain in several different patient populations.^24^, ^25^, ^26^ In patients with migraine, a meta-analysis of three small trials concluded that prophylactic melatonin use reduced the frequency, severity, and duration of migraine compared with placebo but was not superior to amitriptyline.^27^ A recent meta-analysis of four small trials showed reduced severity of irritable bowel syndrome after variable durations of melatonin treatment, although sleep was not improved.^28^ The doses of melatonin used in these studies were variable; there is no consensus as to what constitutes a clinically effective dose. Circadin™ is a licensed product and the data sheet and summary of product characteristics are publicly available. The absolute bioavailability of Circadin™ is assumed to be similar to other melatonin formulations (approximately 15%) but has not been measured. There is delayed absorption of Circadin™ (~ 2 h) compared with melatonin in other formulations (~ 30–60 min). Melatonin given in capsules is very rapidly cleared from the circulation.^29^ Circadin™ is a prolonged-release formulation of melatonin designed to mimic endogenous secretion, and we used the dose licensed for treating primary insomnia (2 mg). We have showed previously that larger doses of Circadin™ result in prolonged elevation of melatonin concentration which may impact on daytime activities.^17^ Most trials do not report on circulating melatonin concentration or daytime drowsiness even when giving high doses.

The average pain intensity and sleep interference scores captured by the BPI decreased during both treatment periods in our trial, but there was no difference between melatonin and placebo treatments, suggesting that participation in the trial had a positive effect regardless of treatment allocation, as has been previously reported.^30^ We did not measure anxiety and depression at baseline. The COVID-19 pandemic may have had an impact on psychological status in our participants, but the BPI interference scores of psychological parameters were similar at baseline in both groups and did not change. Our participants included those with various types of pain syndromes and there were some differences in the types of pain in the two treatment groups. Analysis of the effects of melatonin by pain type was not possible because of small numbers.

Participants taking part in crossover trials are randomised not to a treatment *per se*, but to the sequence in which active drug or placebo is administered, so each person gets the active drug at some point.^31^ Calculation of differences between treatments use within-subject comparisons, so confounding factors are minimised, and as a result, such trials have high power and are statistically efficient.^32^ Crossover trials may create issues with carryover of drug effects, but a 2 mg dose of Circadin™ is eliminated in 12 h and our washout period was a minimum of 4 weeks. We also measured circulating melatonin concentration 12 h after dosing in a subset of participants and found no difference during melatonin and placebo treatment periods, indicating no carryover, unlike with higher doses of Circadin™.^17^ As participants in crossover studies can be in the trial for longer than other trial designs, the withdrawal rate can be a problem, but our dropout rate was less than we had anticipated, with only 23% of participants withdrawing. Finally, crossover trials require participants to be stable for the duration of the trial; for this reason, we required participants to be stable on their current medication for at least 4 weeks prior to recruitment and changes other than to dose were not allowed. Most participants were taking several types of medication (Supplementary Table S1) and there were no obvious differences between the groups.

Although it was a single-centre study, it was adequately powered to detect both a difference in VSH sleep disturbance of 120 and pain intensity score of 2 points after melatonin treatment compared with placebo.^15^ We chose to use sleep disturbance captured by the VSH scale as our primary outcome measure (Supplementary Fig. S6) as it has been widely validated,^16^^,^^17^^,^^33^^,^^34^ and we previously found that VSH sleep disturbance correlated with pain intensity scores in a similar patient population.^14^ The sample size calculation was based on our own data from the local patient population rather than data from other centres from unrelated studies. We used three different validated sleep scales and it is of note that all three sleep scores concurred, in that a significant effect of melatonin was seen after 3 weeks of treatment which was significantly different between treatment periods.

The self-reported sleep scales and pain intensity scoring used here are of course inherently subjective; we also used Actigraph watches which objectively capture sleep data including the time in bed, the number of hours asleep, latency, efficiency, and WASO.^35^ However, much of the captured sleep data seem implausible, with a few participants apparently sleeping for over 20 h in a 24-h period. Inactivity being recorded as sleep was because of extended periods of low activity such that the software was unable to distinguish between actual sleep and inactivity. This has been reported before, resulting in underestimations of latency and overestimation of sleep duration.^35^^,^^36^ However, the use of the device to input pain and fatigue scores twice daily worked well and compliance was very good (98%) as there was an audible alarm to remind participants. This real-time ecological momentary assessment data allowed us to assess overall pain during the study, tracking fluctuations in real time and not being subject to recall bias. Analysis of the daily actual ‘pain now’ scores inputted to the Actigraph watch showed that overall, scores were significantly lower but were very small and clinically not significant during the melatonin treatment period than during placebo treatment, as were daily fatigue scores. However, the self-reported impact of pain and poor sleep quality on quality of life as perceived by the individual may be more important than group data, and meaningful improvements for the individual patient are likely to be different to group differences found in the context of a clinical trial.^37^

This study was a single-centre trial of patients attending a tertiary referral clinic. Patients with chronic pain are usually managed in a primary/community care setting and the participants in our trial represent the most severely affected of these patients. Much of the trial was conducted during the COVID-19 pandemic and therefore required minor changes to the protocol. Although recruitment was put on hold for a few weeks, participants already randomised continued to receive their medication and overall, there was little impact.

In summary, Circadin™ treatment did not improve sleep disturbance in patients with severe chronic pain compared with placebo at 6 weeks, but consistently improved several measures of sleep quality in the shorter term. Melatonin has an excellent safety profile and might benefit individual patients, perhaps during flare-up episodes or when respite is required.

## Authors’ contributions

Study design: HG, SK, RA

Data collection: UO, HG, SK

Data analysis: MC, HG

Drafting the manuscript: HG

Reviewing the manuscript: all authors

## Acknowledgements

We are very grateful to Flynn Pharma Ltd. for the provision of Circadin™ and placebo tablets for this trial. We acknowledge the members of the Trial Steering Group and the Data and Safety Monitoring Committee and the trial participants.

## Declaration of interest

HFG is a trustee and director of the *British Journal of Anaesthesia*.

## Funding

*British Journal of Anaesthesia*/Royal College of Anaesthetists, grant reference WKR0-2017-0043.

## Data availability statement

The raw data supporting the conclusions of this article will be made available by the authors, without undue reservation.

## Patient and public involvement

Patients were involved in the design and dissemination plans of this research and the Trial Steering Group had representation from a member of the public.
