# Supplementary material for: Melatonin treatment has consistent but transient beneficial effects on sleep measures and pain in patients with severe chronic pain: the DREAM–CP randomised controlled trial
Source: Br J Anaesth. 2024 Feb 14;132(4):725–34. doi: 10.1016/j.bja.2024.01.012 (PMC10925889; doi:10.1016/j.bja.2024.01.012)
Supplement: Multimedia component 1 [file mmc1.pptx]

## Slide 1
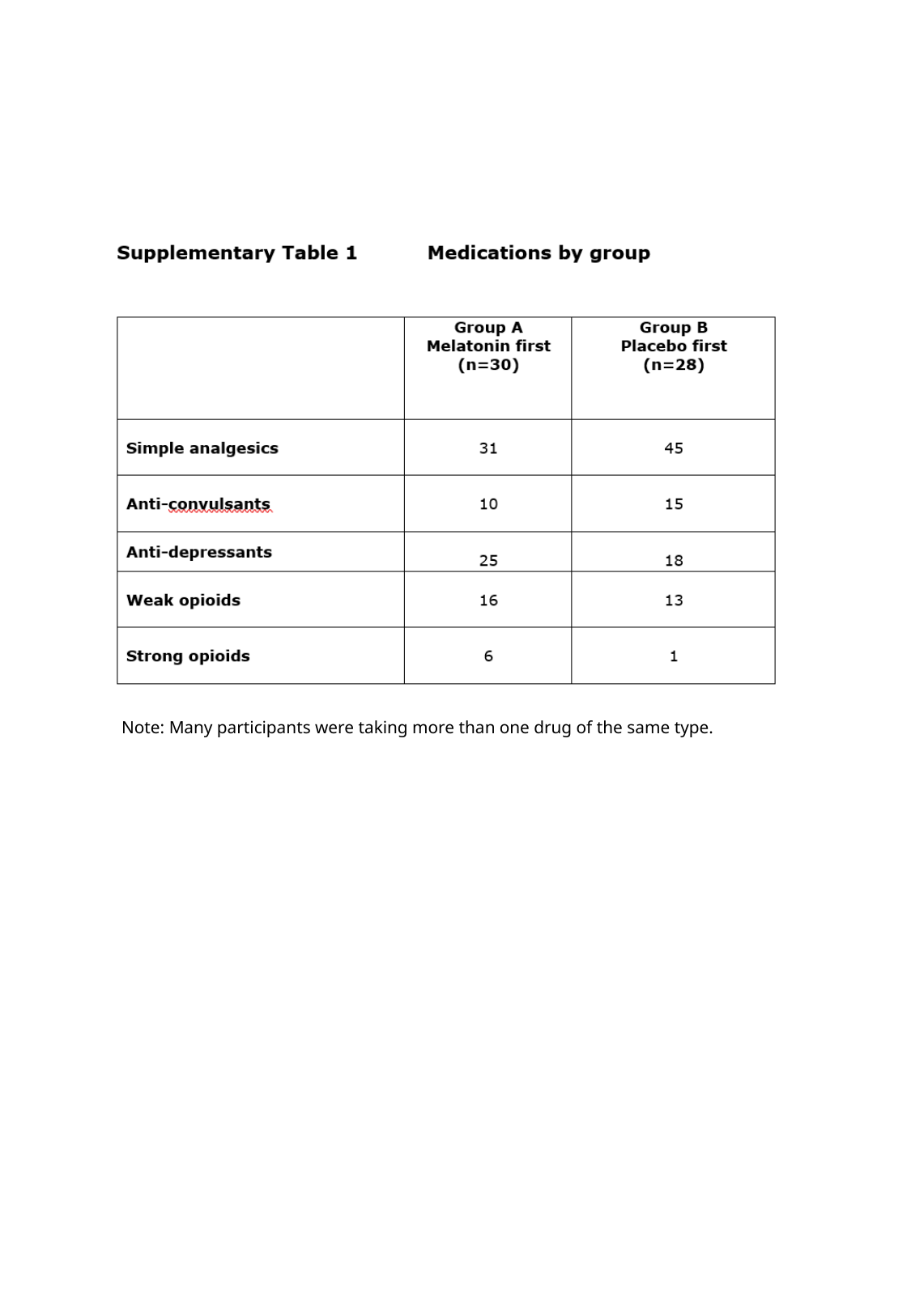

Note: Many participants were taking more than one drug of the same type.

## Slide 2
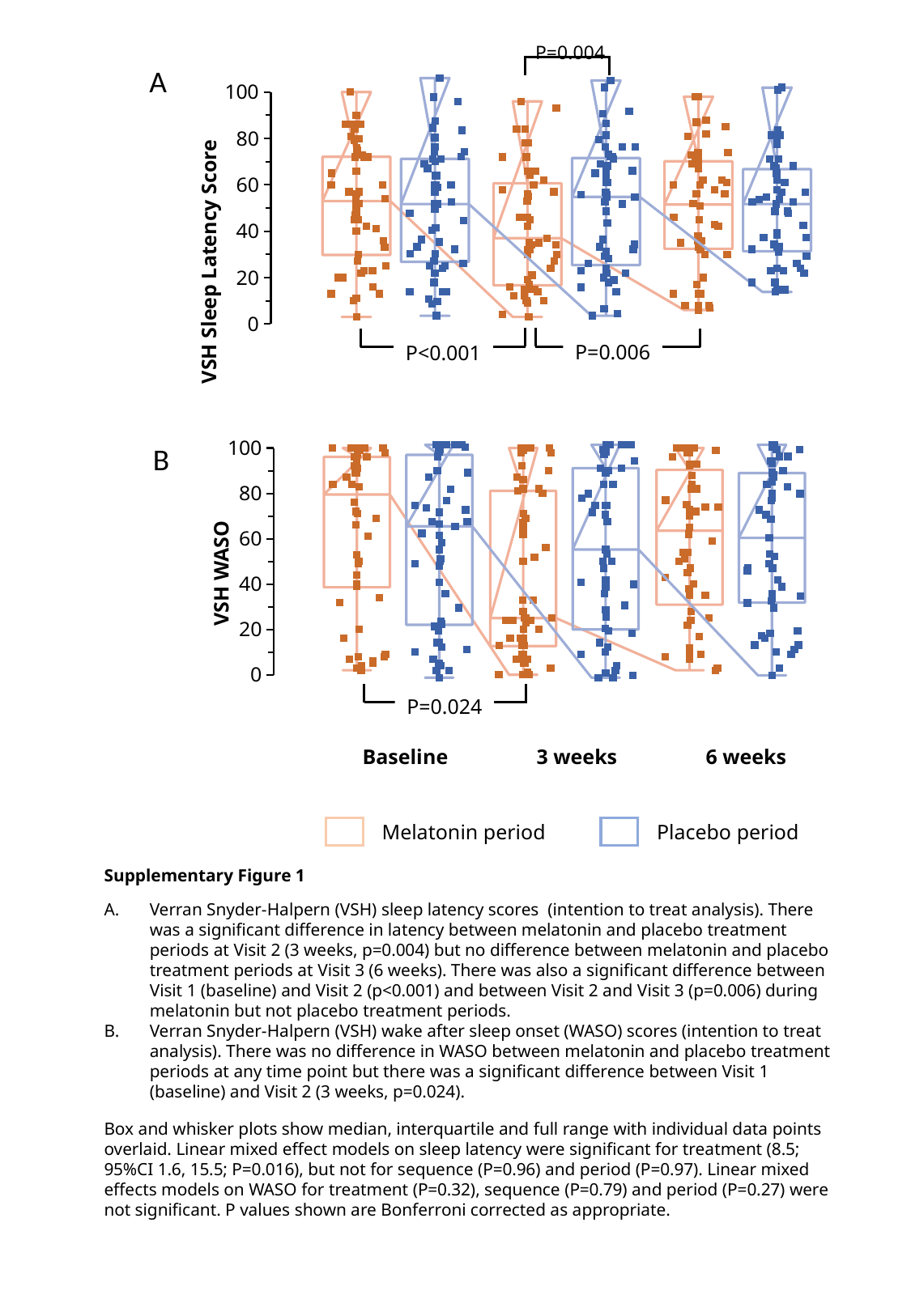

P=0.004
[unsupported chart]
[unsupported chart]
VSH Sleep Latency Score
P=0.006
P<0.001
A
B
[unsupported chart]
[unsupported chart]
VSH WASO
P=0.024
Baseline
3 weeks
6 weeks
Placebo period
Melatonin period
Supplementary Figure 1
Verran Snyder-Halpern (VSH) sleep latency scores (intention to treat analysis). There was a significant difference in latency between melatonin and placebo treatment periods at Visit 2 (3 weeks, p=0.004) but no difference between melatonin and placebo treatment periods at Visit 3 (6 weeks). There was also a significant difference between Visit 1 (baseline) and Visit 2 (p<0.001) and between Visit 2 and Visit 3 (p=0.006) during melatonin but not placebo treatment periods.
Verran Snyder-Halpern (VSH) wake after sleep onset (WASO) scores (intention to treat analysis). There was no difference in WASO between melatonin and placebo treatment periods at any time point but there was a significant difference between Visit 1 (baseline) and Visit 2 (3 weeks, p=0.024).
Box and whisker plots show median, interquartile and full range with individual data points overlaid. Linear mixed effect models on sleep latency were significant for treatment (8.5; 95%CI 1.6, 15.5; P=0.016), but not for sequence (P=0.96) and period (P=0.97). Linear mixed effects models on WASO for treatment (P=0.32), sequence (P=0.79) and period (P=0.27) were not significant. P values shown are Bonferroni corrected as appropriate.

## Slide 3
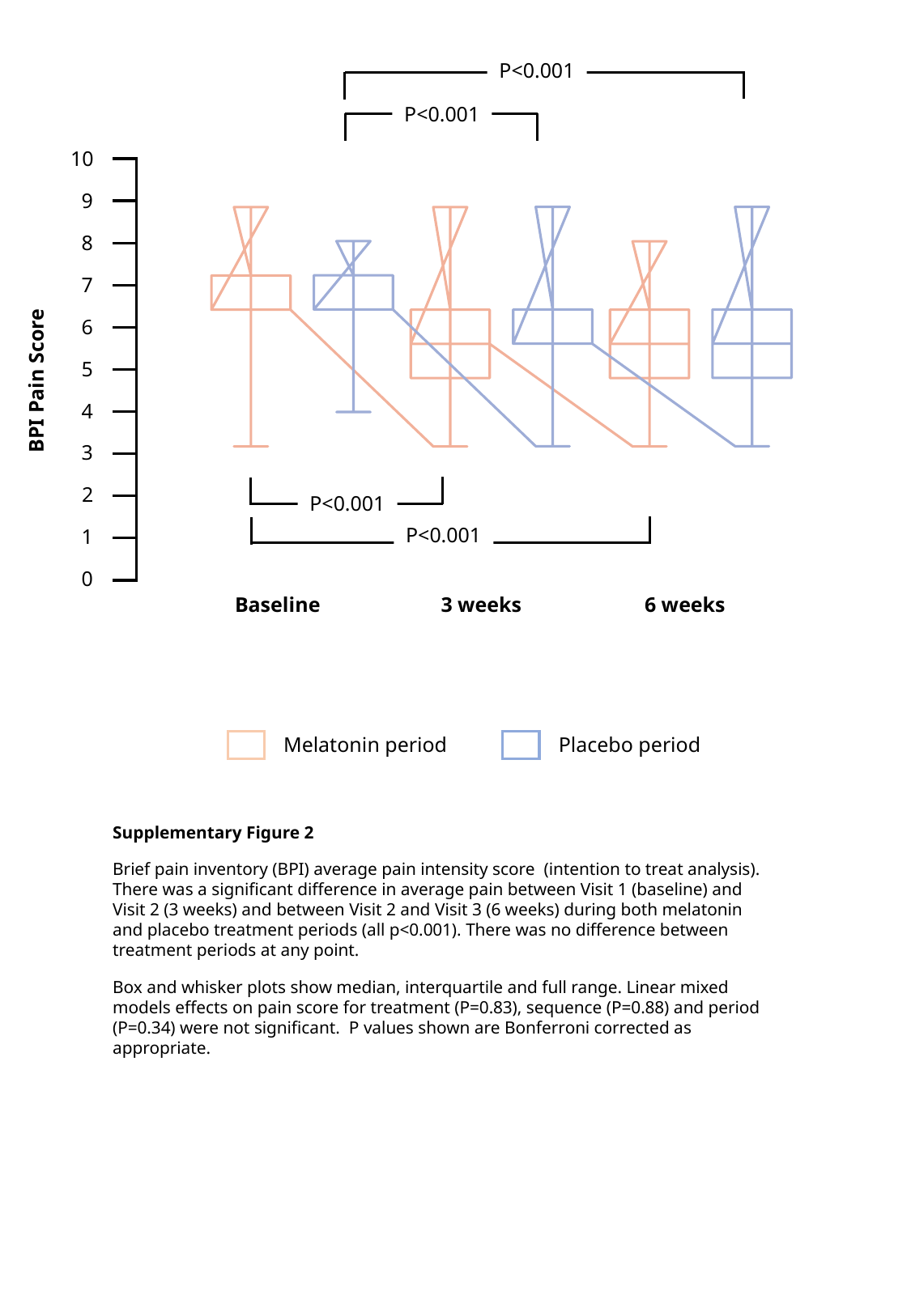

P<0.001
P<0.001
[unsupported chart]
[unsupported chart]
BPI Pain Score
P<0.001
P<0.001
10
9
8
7
6
5
4
3
2
1
0
Baseline
3 weeks
6 weeks
Placebo period
Melatonin period
Supplementary Figure 2
Brief pain inventory (BPI) average pain intensity score (intention to treat analysis). There was a significant difference in average pain between Visit 1 (baseline) and Visit 2 (3 weeks) and between Visit 2 and Visit 3 (6 weeks) during both melatonin and placebo treatment periods (all p<0.001). There was no difference between treatment periods at any point.
Box and whisker plots show median, interquartile and full range. Linear mixed models effects on pain score for treatment (P=0.83), sequence (P=0.88) and period (P=0.34) were not significant. P values shown are Bonferroni corrected as appropriate.

## Slide 4
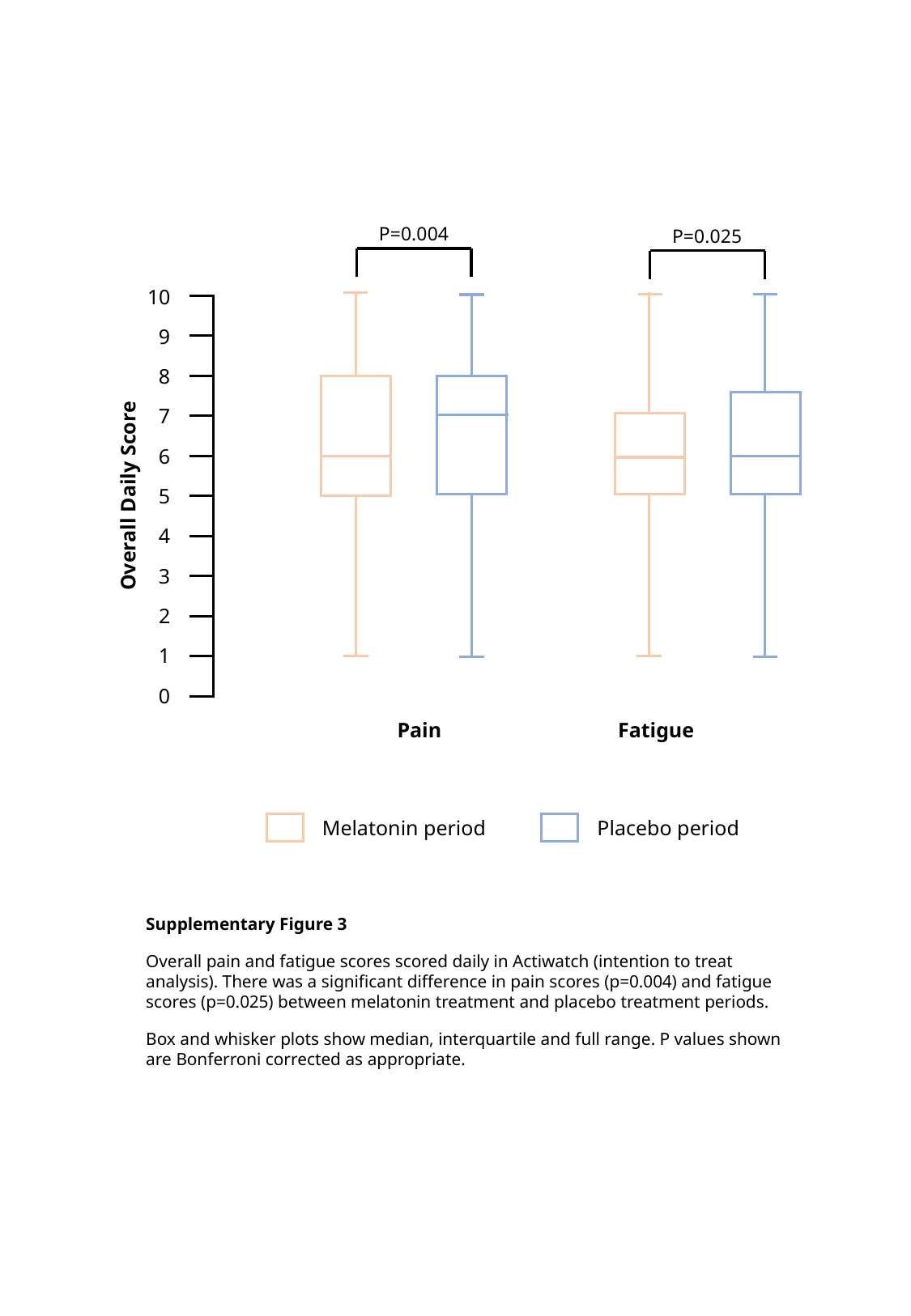

P=0.004
10
9
8
7
6
5
4
3
2
1
0
Overall Daily Score
Fatigue
Pain
P=0.025
Placebo period
Melatonin period
Supplementary Figure 3
Overall pain and fatigue scores scored daily in Actiwatch (intention to treat analysis). There was a significant difference in pain scores (p=0.004) and fatigue scores (p=0.025) between melatonin treatment and placebo treatment periods.
Box and whisker plots show median, interquartile and full range. P values shown are Bonferroni corrected as appropriate.

## Slide 5
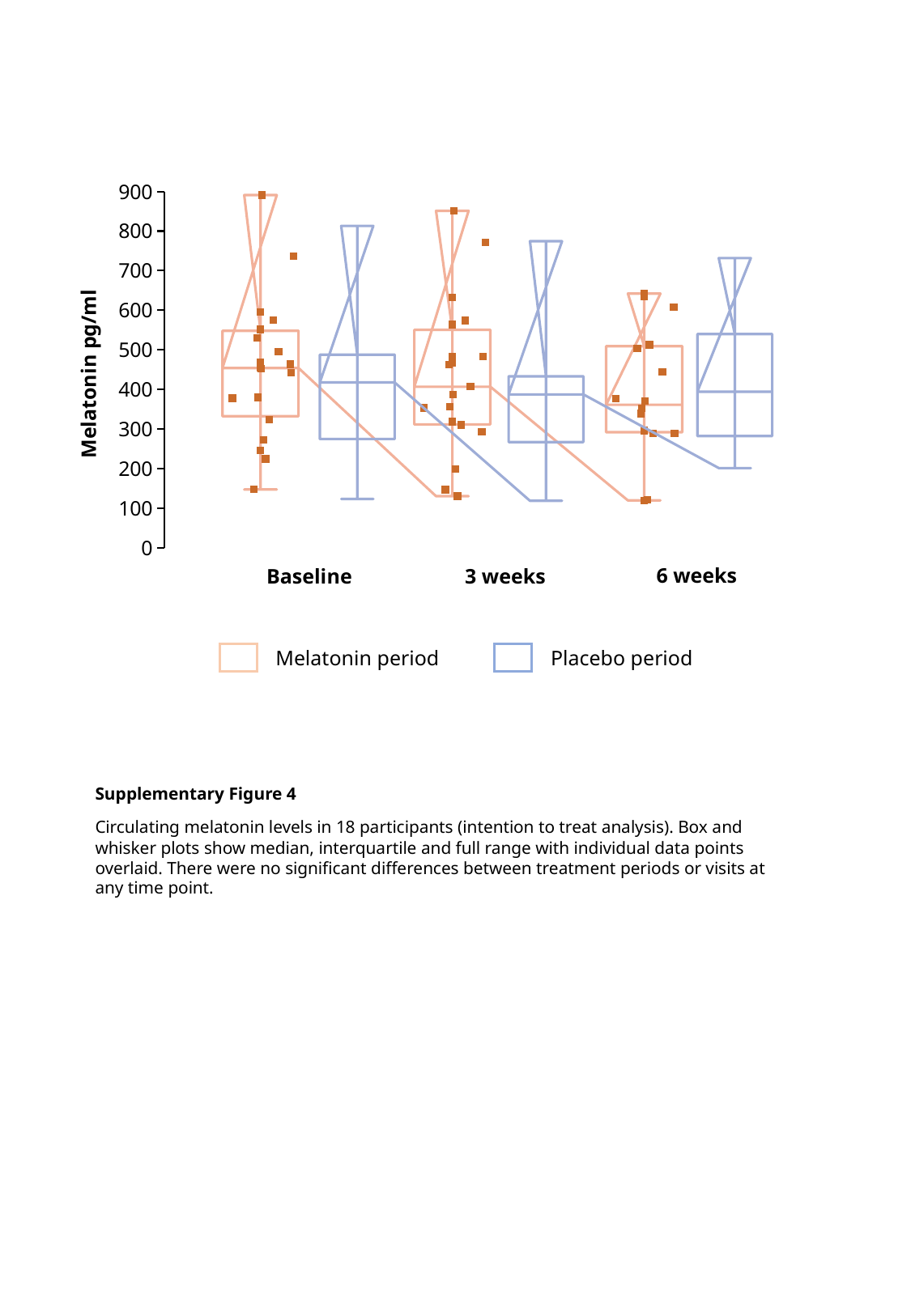

[unsupported chart]
[unsupported chart]
Melatonin pg/ml
6 weeks
Baseline
3 weeks
Placebo period
Melatonin period
Supplementary Figure 4
Circulating melatonin levels in 18 participants (intention to treat analysis). Box and whisker plots show median, interquartile and full range with individual data points overlaid. There were no significant differences between treatment periods or visits at any time point.

## Slide 6
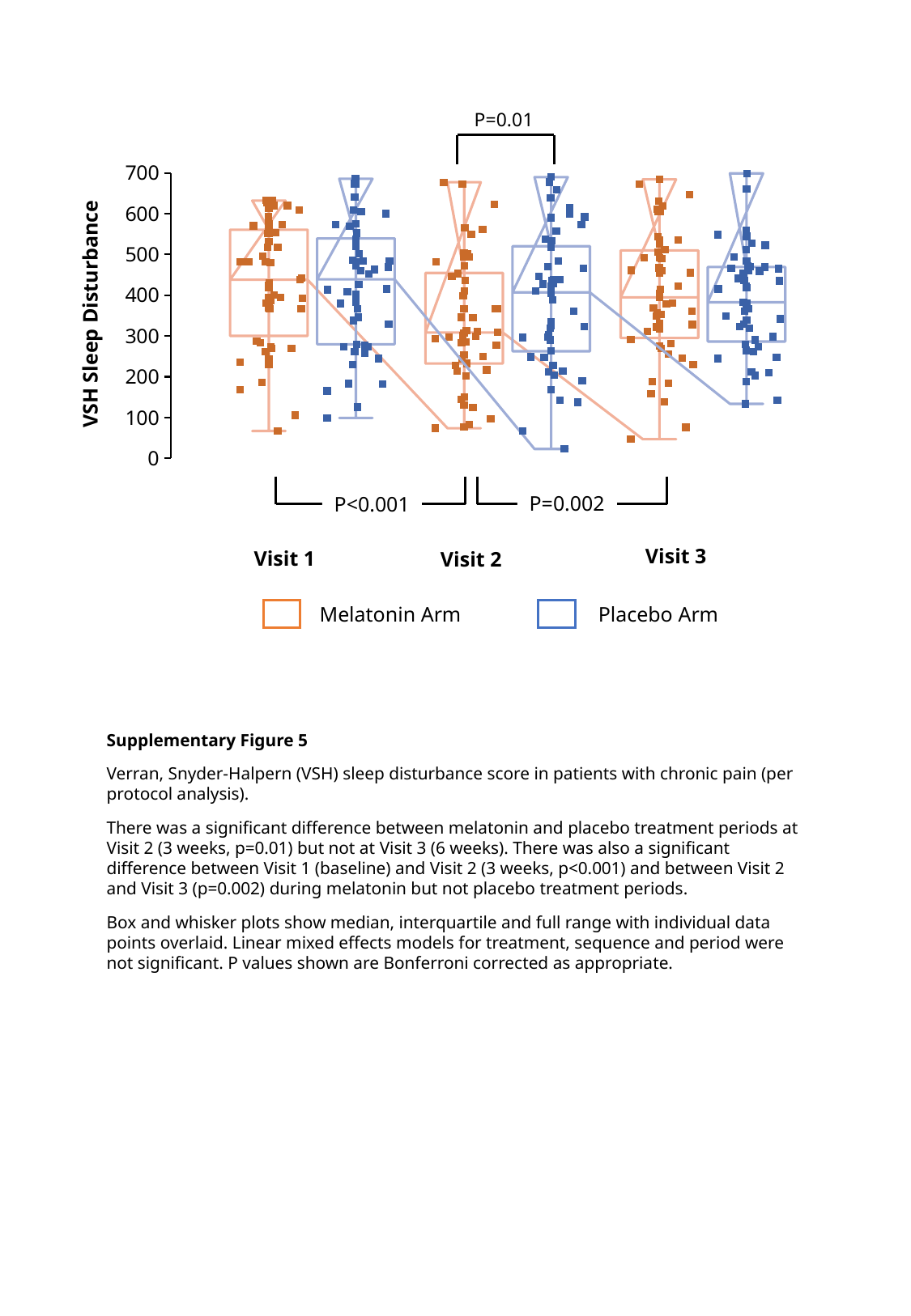

P=0.01
[unsupported chart]
[unsupported chart]
VSH Sleep Disturbance
P=0.002
P<0.001
Visit 3
Visit 1
Visit 2
Placebo Arm
Melatonin Arm
Supplementary Figure 5
Verran, Snyder-Halpern (VSH) sleep disturbance score in patients with chronic pain (per protocol analysis).
There was a significant difference between melatonin and placebo treatment periods at Visit 2 (3 weeks, p=0.01) but not at Visit 3 (6 weeks). There was also a significant difference between Visit 1 (baseline) and Visit 2 (3 weeks, p<0.001) and between Visit 2 and Visit 3 (p=0.002) during melatonin but not placebo treatment periods.
Box and whisker plots show median, interquartile and full range with individual data points overlaid. Linear mixed effects models for treatment, sequence and period were not significant. P values shown are Bonferroni corrected as appropriate.

## Slide 7
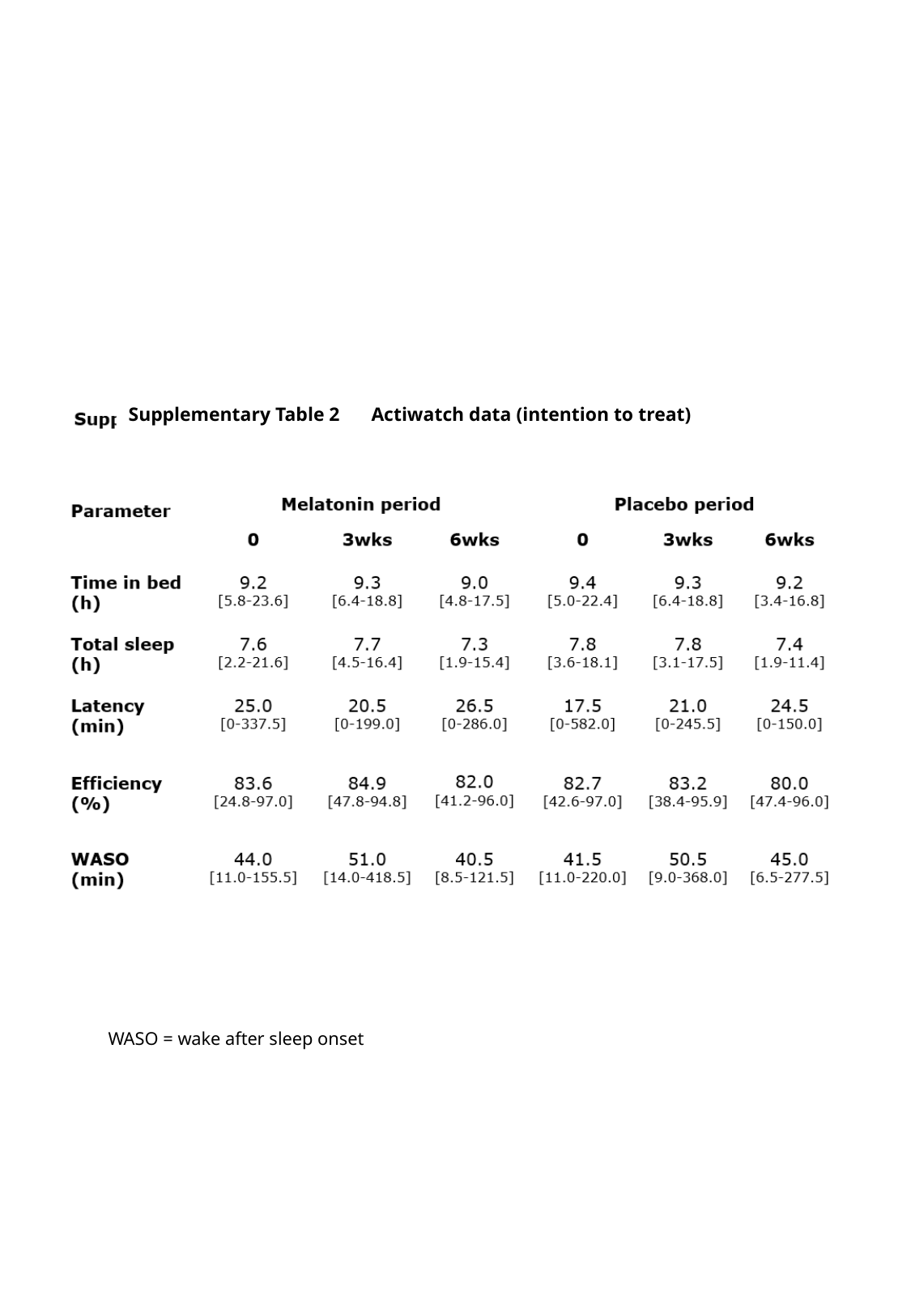

Supplementary Table 2 	Actiwatch data (intention to treat)
WASO = wake after sleep onset

## Slide 8
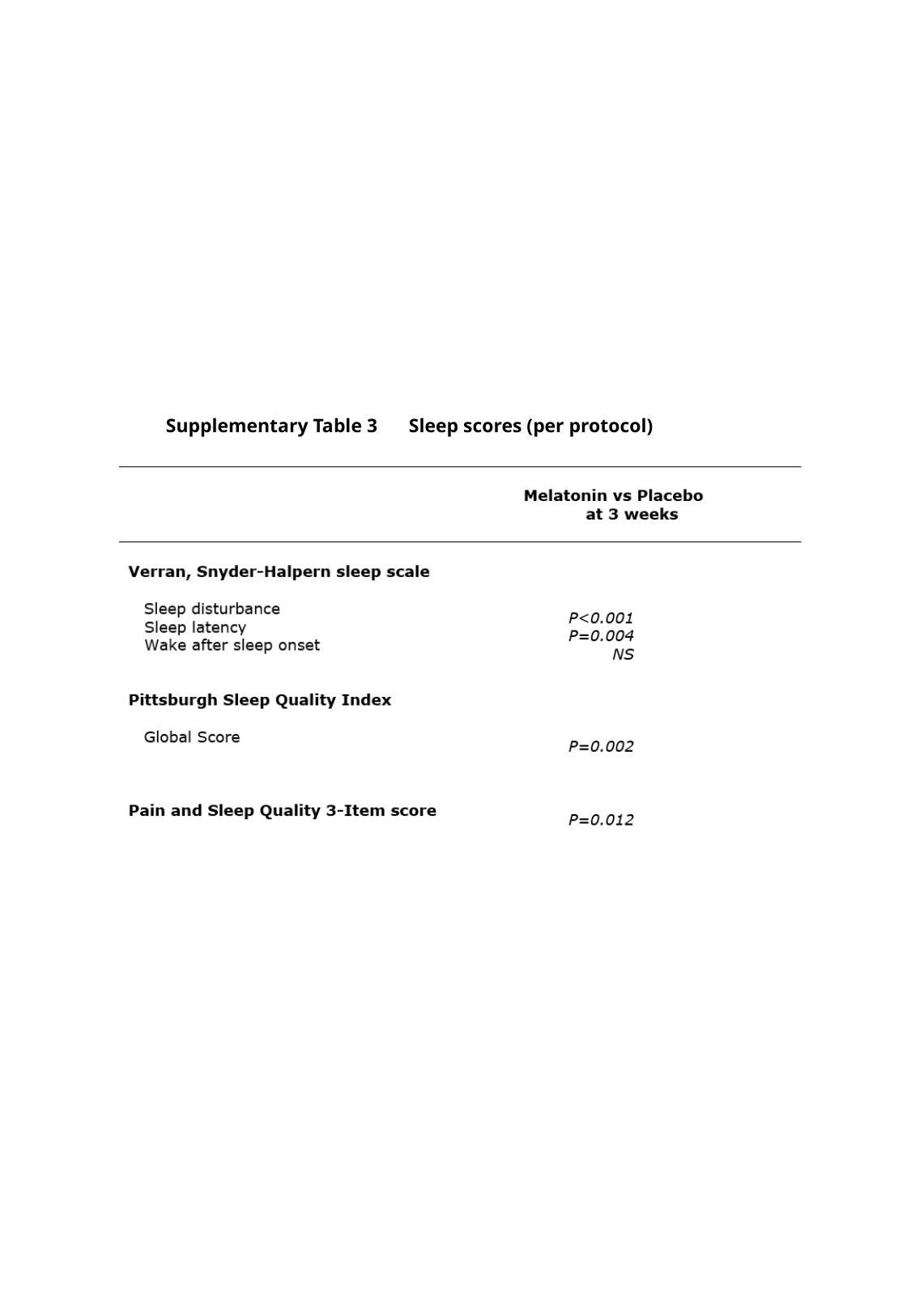

Supplementary Table 3 	Sleep scores (per protocol)

## Slide 9
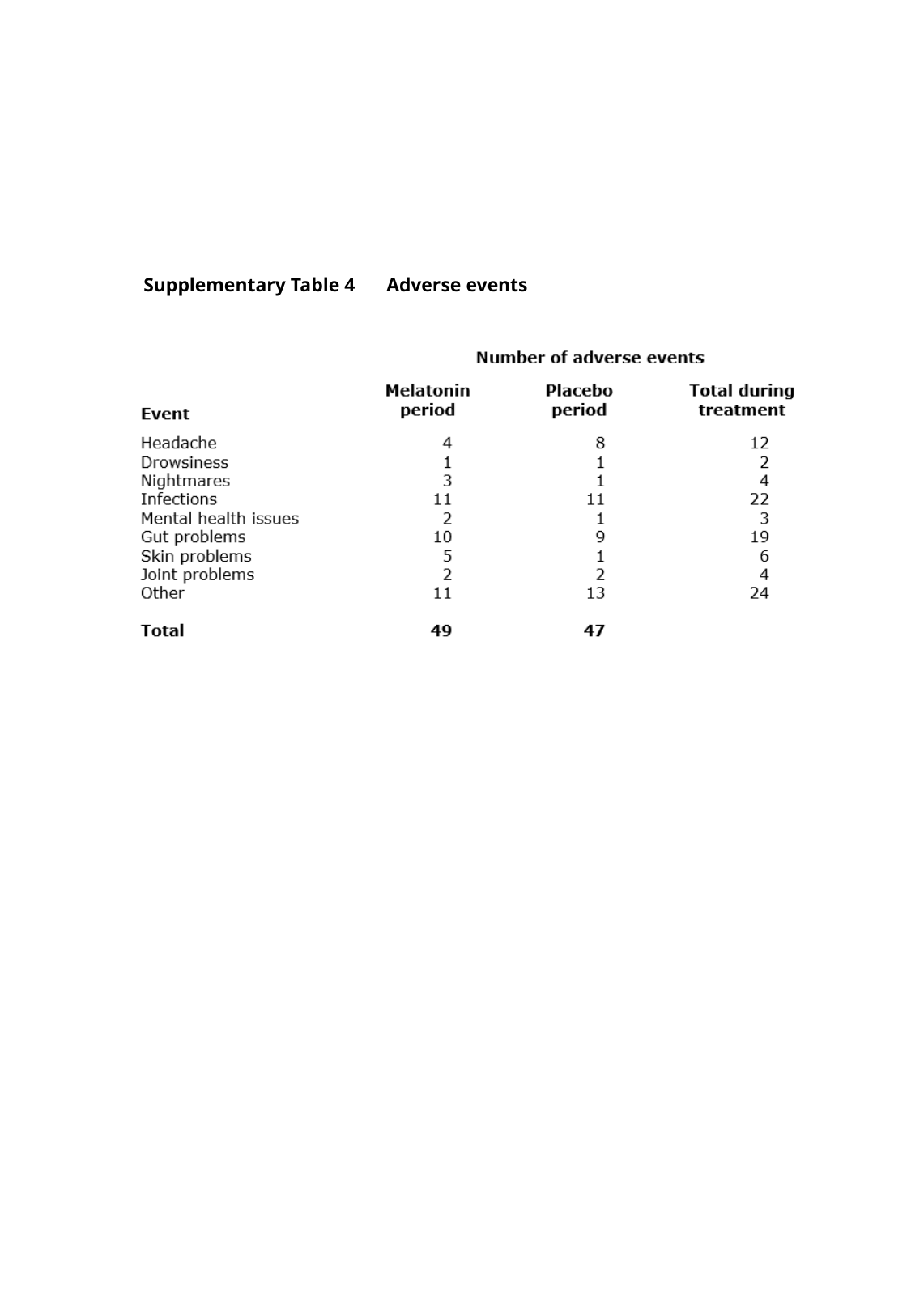

Supplementary Table 4 	Adverse events

## Slide 10
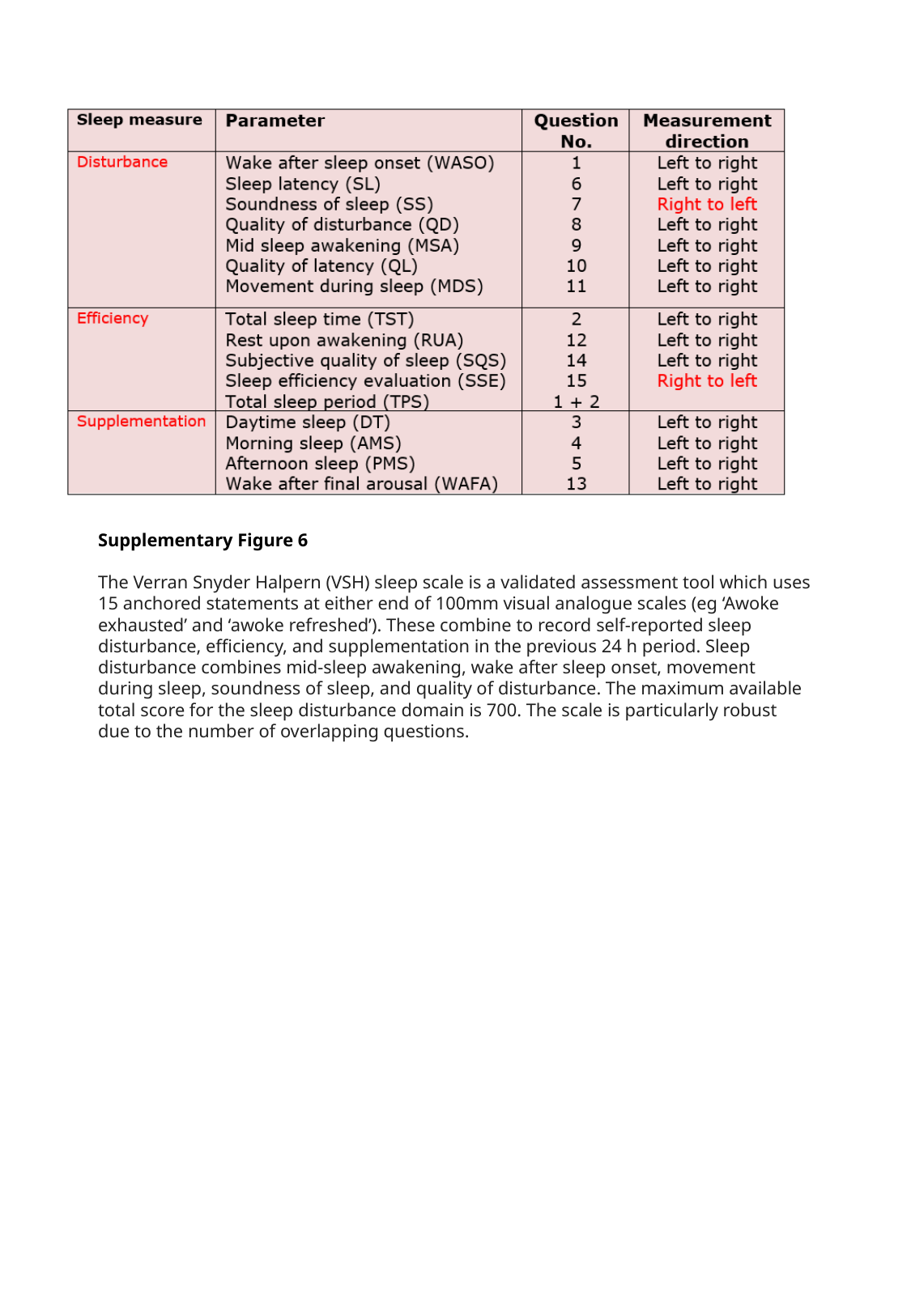

Supplementary Figure 6
The Verran Snyder Halpern (VSH) sleep scale is a validated assessment tool which uses 15 anchored statements at either end of 100mm visual analogue scales (eg ‘Awoke exhausted’ and ‘awoke refreshed’). These combine to record self-reported sleep disturbance, efficiency, and supplementation in the previous 24 h period. Sleep disturbance combines mid-sleep awakening, wake after sleep onset, movement during sleep, soundness of sleep, and quality of disturbance. The maximum available total score for the sleep disturbance domain is 700. The scale is particularly robust due to the number of overlapping questions.
